# LncRNA CRNDE promotes cell proliferation, invasion and migration by competitively binding miR-384 in papillary thyroid cancer

**DOI:** 10.18632/oncotarget.22819

**Published:** 2017-11-30

**Authors:** Honggang Sun, Liqin He, Lan Ma, Tao Lu, Jianguo Wei, Kejie Xie, Xingmu Wang

**Affiliations:** ^1^ Clinical Laboratory Center, Shaoxing People's Hospital, Shaoxing Hospital of Zhejiang University, Shaoxing, Zhejiang Province, China; ^2^ Department of Rehabilitation, Shaoxing People's Hospital, Shaoxing Hospital of Zhejiang University, Shaoxing, Zhejiang Province, China; ^3^ Department of Pathology, Shaoxing People's Hospital, Shaoxing Hospital of Zhejiang University, Shaoxing, Zhejiang Province, China

**Keywords:** CRNDE, papillary thyroid cancer, miR-384, pleiotrophin, progression

## Abstract

Thyroid cancer is one of the most prevalent endocrine neoplasm. The present study examined the effects of Colorectal Neoplasia Differentially Expressed (CRNDE) on the progression of papillary thyroid cancer (PTC), and explored the underlying molecular mechanisms. Quantitative real-time PCR was used to detect CRNDE, miR-384 and pleiotrophin (PTN) mRNA expression. Western blot was used to measure PTN protein levels. Cell proliferation, cell growth, cell invasion and migration of PTC cells were determined by CCK-8, colony formation, transwell invasion and migration assays, respectively. CRNDE was up-regulated in PTC tissues and cell lines. Overexpression of CRNDE promoted BCPAP cell proliferation, invasion and migration, while knock-down of CRNDE suppressed K1 cell proliferation, invasion and migration. CRNDE negatively regulated the expression of miR-384 in PTC cells, which was further confirmed by luciferase reporter assay. MiR-384 was down-regulated and inversely correlated with CRNDE expression in PTC tissues. MiR-384 suppressed cell proliferation, invasion and migration in PTC cells, and enforced expression of miR-384 attenuated the oncogenic effects of CRNDE in PTC cells. PTN was predicted as a downstream target of miR-384, which was confirmed by luciferase reporter assay, and PTN was up-regulated in PTC tissues, and was negatively correlated with miR-384 expression and positively correlated with CRNDE expression in PTC tissues. In summary, our results suggested that the CRNDE/miR-384/PTN axis may play an important role in the regulation of PTC progression, which provides us with new insights into understanding the PTC.

## INTRODUCTION

Thyroid cancer is one of the most prevalent endocrine neoplasm with new cases of ∼300,000 worldwide, and the median age at diagnosis for thyroid cancer is ∼50 years old and the annual death is ∼40,000 [1]. Papillary thyroid cancer (PTC) accounts for 80% of all thyroid cancers, and is the most common histological type [2]. The 5-year survival rate for PTC patients is more than 90% [3]. However, ∼20% of the PTC patients had lymph node metastasis and the patients undergoing total thyroidectomy are with a regional recurrence of ∼10% [3]. Currently, the gold standard for diagnosis is needle aspiration biopsy, but the predicative value for recurrence is rather limited, due to the limited understanding of the mechanisms underlying PTC progression [4]. In this regard, investigating the molecular mechanism underlying PTC progression is extremely imperative and necessary.

The long non-coding RNAs (lncRNAs) are a class of non-coding RNA longer than 200 nucleotides, and can not be translated into proteins [5]. LncRNAs involved in may key biological processes, such as transcription regulation, epigenetic regulation, chromatin remodeling and mRNA processing [6]. Growing evidence has showed that lncRNAs play important roles in a wide range of human cancers [7, 8]. The lncRNA, Colorectal Neoplasia Differentially Expressed (CRNDE) was first identified in colorectal adenomas and adenocarcinomas, and the CRNDE locus spans ∼10 kb and comprises five core exons with an additional exon that is infrequently present in transcripts [9]. Apart from the the oncogenic role of CRNDE in colorectal adenomas and adenocarcinomas, the dysregulations of CRNDE were also found in other types of cancers including liver cancer [10], gastric cancer [11], gallbladder carcinoma [12], breast cancer [13], glioma [14]. Recently, CRNDE was found to be up-regulated in the PTC tissues [15]. However, the functional role of CRNDE in the PTC progression is unclear.

MicroRNAs (miRNAs) are a class of endogenous, small non-coding RNAs containing about 22 nucleotides, and they can degrade mRNA molecules or inhibit the translation by targeting the 3′ untranslated region (3'UTR) of the targeted genes [16]. MiRNAs have been found to involve in various biological actions, such as tumorigenesis and cancer metastasis, and the dysregulation of miRNAs has been demonstrated in a large number of studies about various types of cancers [17]. In the PTC, miRNAs, such as miR-96 [18], miR-146-5p [19], and miR-155 [20], were found to promote the tumor growth of PTC ; while miRNAs, such as miR-150 [21], miR-497 [22], and miR-613 [23] inhibited cell proliferation, invasion/migration of PTC cells. Recently, miR-384 was found to involve in the cancer progression of glioma [24], hepatocellular carcinoma [25], and colorectal cancer [26]. However, up to date, the functional role of miR-384 in PTC was not examined.

In the present study, we firstly examined the *in vitro* functional role of CRNDE in PTC cell lines, and the interaction between CRNDE and miR-384 was predicted by bioinformatics analysis and confirmed by the luciferase reporter assay. In addition, the effects of miR-384 on PTC cells proliferation, invasion/migration were examined, and the downstream targets of miR-384 was also explored. The present study aimed to elucidate the effects of CRNDE, miR-384 and the downstream targets of miR-384 on the progression of PTC.

## RESULTS

### CRNDE is up-regulated in PTC tissues and PTC cell lines

**Figure 1:**
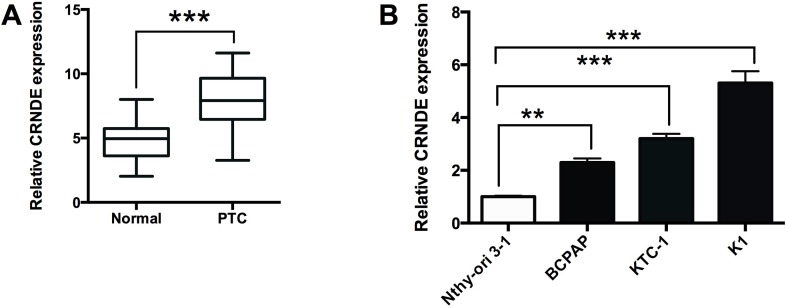
CRNDE is up-regulated in PTC tissues and PTC cell lines **(A)** Analysis of 40 paired tumor tissue samples (adjacent non-tumor tissue samples and tumor tissues) showed that the expression of CRNDE was increased in tumor tissues (PTC) compared with adjacent normal tissues (N = 40), ^***^P<0.001. **(B)** CRNDE expression was up-regulated in PTC cells (BCPAP, KTC-1 and K1 cells) compared with normal PT cells (Nthy-ori 3-1) (N = 4), ^**^P<0.01, ^***^P<0.001.

To confirm the expression of CRNDE in PTC tissues, we performed qRT-PCR experiments to determine the expression of CRNDE in 40 adjacent normal thyroid tissues and 40 PTC tissues, and CRNDE in the PTC tissues was up-regulated compared with adjacent normal tissues (Figure 1A). The expression of CRNDE was also detected in normal thyroid cells (Nthy-ori 3-1) and PTC cell lines (BCPAP, KTC-1 and K1 cells), and the expression of CRNDE in PTC cells were significantly higher than that in Nthy-ori 3-1 cells (Figure 1B).

### Effects of CRNDE overexpression/suppression on the proliferation and invasion/migration in PTC cells

**Figure 2:**
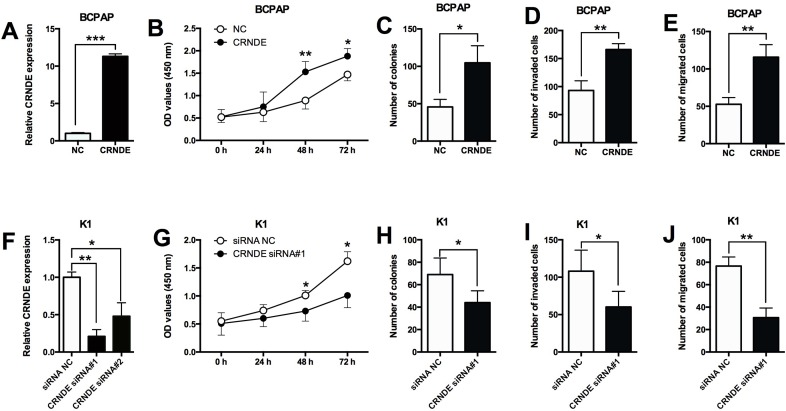
Effects of CRNDE overexpression/suppression on the proliferation and invasion/migration in PTC cells **(A)** BCPAP cells transfected with CRNDE-overexpressing vector showed a dramatically increased expression of CRNDE compared with empty vector. **(B)** CRNDE overexpression in BCPAP cells promoted cell proliferation compared with control group (NC) as measured by CCK-8 assay. **(C)** BCPAP cells transfected CRNDE overexpressing vector showed an increased growth ability compared with control group (NC) as measured by colony formation assay. **(D)** Overexpression of CRNDE increased the number of invaded BCPAP cells compared with control group (NC) as measured by transwell invasion assay. **(E)** BCPAP cell transfected with CRNDE overexpressing vector had an increase in the migrated cells compared with control group (NC) as measured by transwell migration assay. **(F)** K1 cells transfected with CRNDE siRNAs showed a decreased expression of CRNDE compared with scrambled siRNA transfection. **(G)** CRNDE suppression in K1 cells inhibited cell proliferation compared with control group (siRNA NC) as measured by CCK-8 assay. **(H)** K1 cells transfected with CRNDE siRNA showed a decreased growth ability compared with control group (siRNA NC) as measured by colony formation assay. **(I)** Suppression of CRNDE decreased the number of invaded K1 cells compared with control group (siRNA NC) as measured by transwell invasion assay. **(J)** Suppression of CRNDE in K1 cells inhibited cell migration compared with control group (NC) as measured by transwell migration assay. N = 4, ^*^P<0.05, ^**^P<0.01, ^***^P<0.001.

To further look into the functional actions of CRNDE in PTC, we performed a series of *in vitro* assays including CCK-8, colony formation, transwell invasion and migration assays in the BCPAP and K1 cells. The up-regulation of CRNDE was achieved by transfecting the BCPAP cells with CRNDE overexpressing vector (pcDNA3.1-CRNDE) (Figure 2A). The overexpressing effects of CRNDE were examined in BCPAP cells, as shown in Figure 2, CRNDE overexpression by transfecting BCPAP cells with CRNDE overexpression vectors significantly promoted cell proliferation (Figure 2B), increased the number of colonies (Figure 2C), and also increased the number of invaded cells (Figure 2D) and migrated cells (Figure 2E). On the other hand, the down-regulation of CRNDE was achieved by transfecting the K1 cells with CRNDE siRNAs (CRNDE siRNA#1 and CRNDE siRNA#2), and we found that CRNDE siRNA#1 was more effective in suppressing the expression of CRNDE than CRNDE siRNA#2 (Figure 2F), thus, CRNDE siRNA#1 was used for further studies. The knock-down effects of CRNDE were examined in K1 cells, CRNDE knock-down by transfecting K1 cells with CRNDE siRNA#1 significantly suppressed cell proliferation (Figure 2G), decreased the number of colonies (Figure 2H), and also suppressed the number of invaded cells (Figure 2I) and migrated cells (Figure 2J).

### MiR-384 is a target of CRNDE

**Figure 3:**
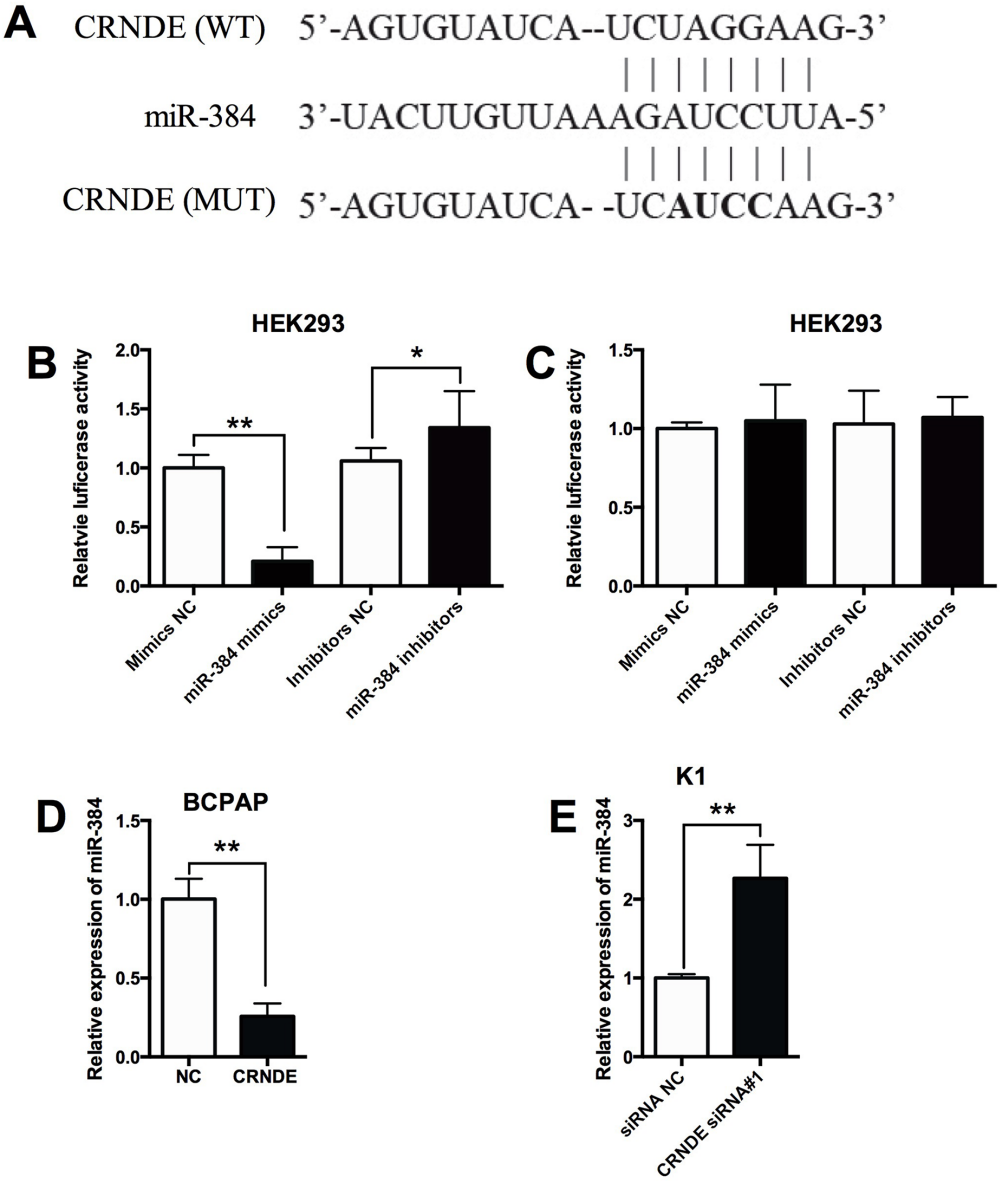
MiR-384 is a target of CRNDE **(A)** The seed sequence of miR-384 matched the sequence of CRNDE gene. The sequences include the wild type (WT) and mutated (MUT) target sites, the mature miR-384 and the target sequence bound by miR-384. **(B)** Luciferase report assay of HEK293 cells co-transfected with CRNDE reporter plasmid containing WT sequences and miR-384 mimics or miR-384 inhibitors, or their corresponding negative controls i.e. mimics NC and inhibitors NC, and luciferase reporter activity was suppressed by miR-384 mimics transfection and increased by miR-384 inhibitors transfection. **(C)** Luciferase report assay of HEK293 cells co-transfected with CRNDE reporter plasmid containing MUT sequences and miR-384 mimics or miR-384 inhibitors, or their corresponding negative controls, and luciferase reporter activity was not affected by miR-384 mimics transfection and miR-384 inhibitors transfection. **(D)** The BCPAP cells transfected with CRNDE overexpressing vector showed a decreased expression of miR-384 in BCPAP cells compared with control group (NC). **(E)** The K1 cells transfected with CRNDE siRNA showed an increased expression of miR-384 compared with control group (siRNA NC). N = 4, ^*^P<0.05, ^**^P<0.01.

As lncRNAs were found to have interaction with miRNAs, the predicted miRNAs that interacts with CRNDE was analyzed by the starbase software, and we found that miR-384 was a potential target of CRNDE (Figure 3A). In order to further confirm the interaction between CRNDE and miR-384, the wild type CRNDE and mutated CRNDE reporter vectors were constructed to perform the luciferase reporter assay. As shown in Figure 3B, miR-384 mimics transfection reduced the luciferase activity of wild type CRNDE reporter vector, while miR-384 inhibitors transfection enhanced the luciferase activity of wild type CRNDE reporter vector; while miR-384 mimics transfection or miR-384 inhibitors transfection had no significant effect on the luciferase activity of mutated CRNDE reporter vector (Figure 3C). In addition, overexpression of CRNDE in BCPAP cells suppressed the expression of miR-384 compared with control group (Figure 3D); knock-down of CRNDE in K1 cells increased the expression of miR-384 compared with control group (Figure 3E).

### Effects of miR-384 overexpression/suppression on the proliferation and invasion/migration in PTC cells

**Figure 4:**
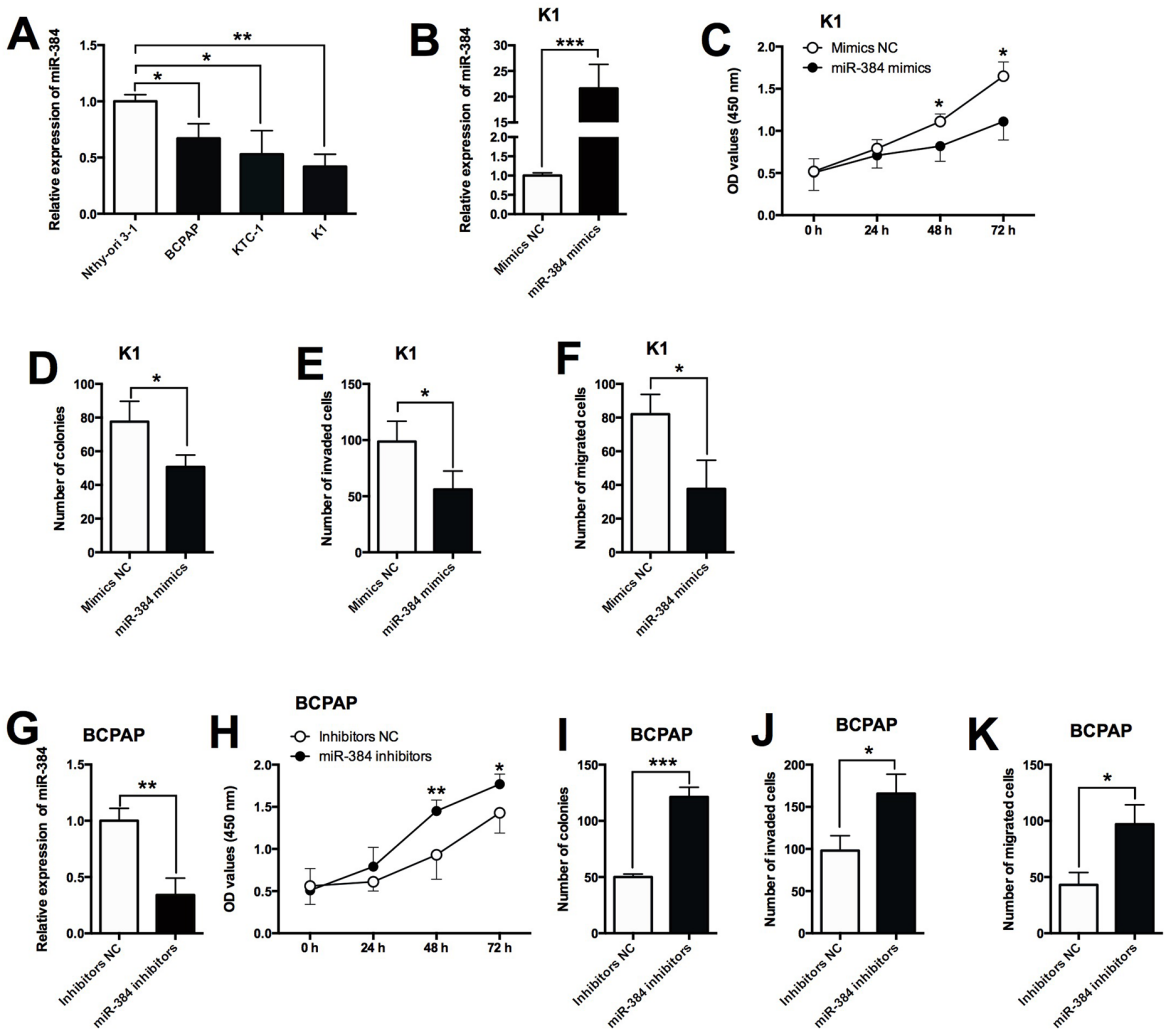
Effects of miR-384 overexpression/suppression on the proliferation and invasion/migration in PTC cells **(A)** MiR-384 expression was down-regulated in PTC cells compared with normal PT cells. **(B)** K1 cells transfected with miR-384 mimics showed a dramatically increased expression of miR-384 compared with empty vector. **(C)** MiR-384 mimics transfection in K1 cells suppressed cell proliferation compared with control group (Mimics NC) as measured by CCK-8 assay. **(D)** K1 cells transfected with miR-384 mimics showed a decreased growth ability compared with control group (Mimics NC) as measured by colony formation assay. **(E)** Overexpression of miR-384 decreased the number of invaded K1 cells compared with control group (Mimics NC) as measured by transwell invasion assay. **(F)** K1 cell transfected with miR-384 mimics had a decrease in the migrated cells compared with control group (Mimics NC) as measured by transwell migration assay. **(G)** BCPAP cells transfected with miR-384 inhibitors showed a decreased expression of miR-384 compared with inhibitors NC transfection. **(H)** MiR-384 down-regulation in BCPAP cells increased cell proliferation compared with control group (Inhibitors NC) as measured by CCK-8 assay. **(I)** BCPAP cells transfected with miR-384 inhibitors showed an increased growth ability compared with control group (Inhibitors NC) as measured by colony formation assay. **(J)** Suppression of miR-384 increased the number of invaded BCPAP cells compared with control group (Inhibitors NC) as measured by transwell invasion assay. **(K)** Suppression of miR-384 in BCPAP cells inhibited cell migration compared with control group (NC) as measured by transwell migration assay. N = 4, ^*^P<0.05, ^**^P<0.01, ^***^P<0.001.

The expression of miR-384 in normal thyroid cells and PTC cell lines was determined by qRT-PCR, and miR-384 was down-regulated in PTC cell lines compared with Nthy-ori 3-1 cells (Figure 4A). To further look into the functional actions of miR-384 in PTC, we performed a series of *in vitro* assays including CCK-8, colony formation, transwell invasion and migration assays in the BCPAP and K1 cells. The up-regulation of miR-384 was achieved by transfecting the K1 cells with miR-384 mimics (Figure 4B), and miR-384 mimics transfection in K1 cells significantly suppressed cell proliferation (Figure 4C), reduced the number of colonies (Figure 4D), and also reduced the number of invaded cells (Figure 4E) and migrated cells (Figure 4F). On the other hand, the down-regulation of miR-384 was achieved by transfecting the BCPAP cells with miR-384 inhibitors (Figure 4G), and the knock-down effects of miR-384 were examined in BCPAP cells, and miR-384 inhibitors transfection in BCPAP cells significantly enhanced cell proliferation (Figure 4H), increased the number of colonies (Figure 4I), and also increased the number of invaded cells (Figure 4J) and migrated cells (Figure 4K).

### MiR-384 is down-regulated and inversely correlated with CRNDE expression in PTC tissues

**Figure 5:**
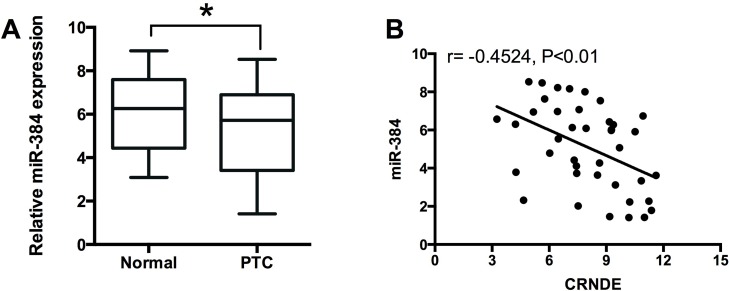
MiR-384 is down-regulated and inversely correlated with CRNDE expression in PTC tissues **(A)** Analysis of 40 paired tumor tissue samples (adjacent non-tumor tissue samples and tumor tissues) showed that the expression of miR-384 was decreased in tumor tissues (PTC) compared with adjacent normal tissues (n = 40), ^*^P<0.05. **(B)** Spearman correlation analysis showed that miR-384 was inversely correlated with CRNDE in PTC tissues, r = -0.4524, P<0.01.

To examine the expression of miR-384 in PTC tissues, we performed qRT-PCR experiments to determine the expression of miR-384 in 40 adjacent normal thyroid tissues and 40 PTC tissues, and miR-384 in the PTC tissues was down-regulated compared with adjacent normal tissues (Figure 5A). The Spearman correlation analysis showed that miR-384 expression was inversely correlated with CRNDE expression in PTC tissues (Figure 5B).

### The effects of CRNDE in PTC cells is dependent on miR-384

**Figure 6:**
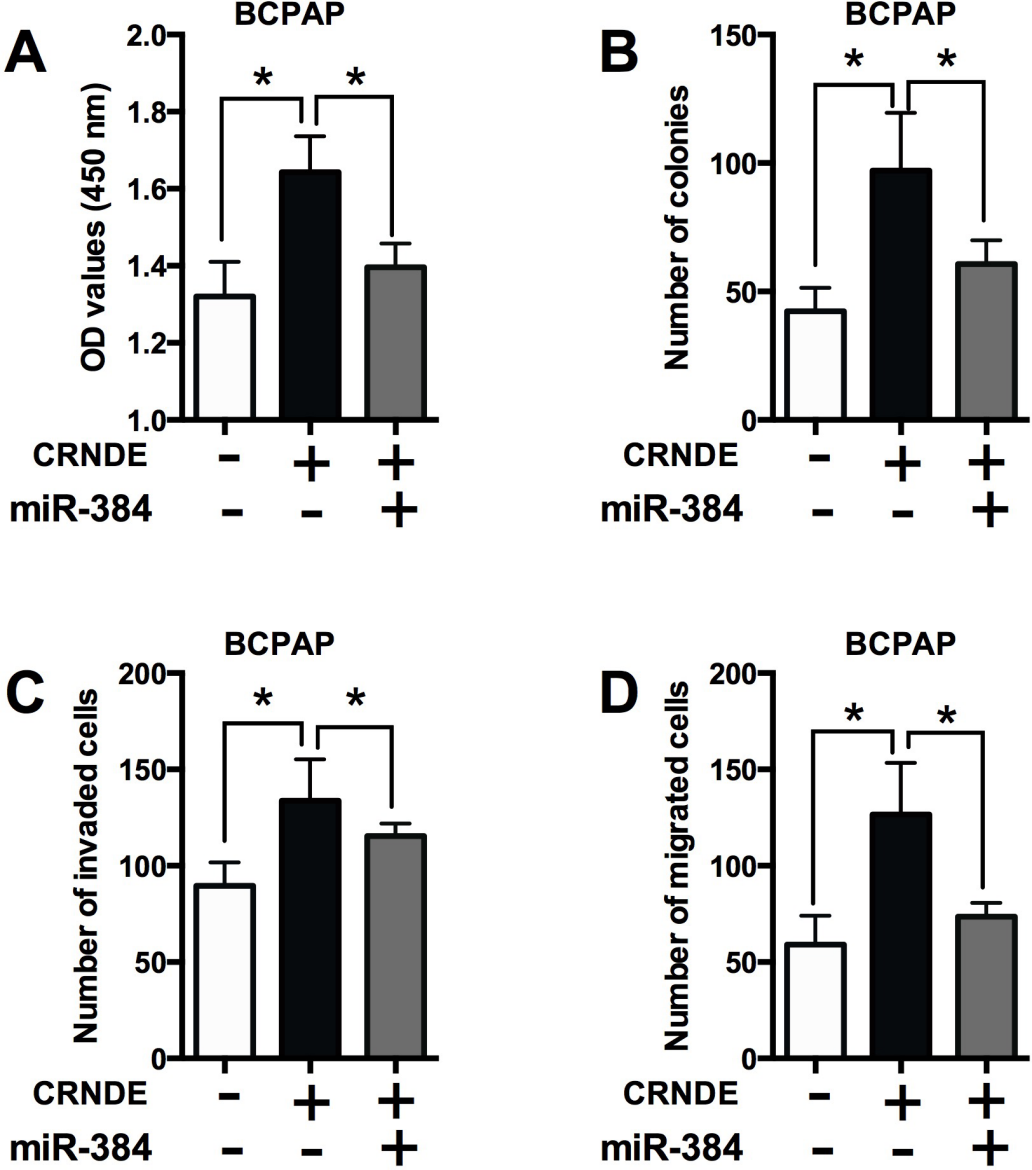
The effects of CRNDE in PTC cells is dependent on miR-384 **(A)** CCK-8 assay analysis was performed to examine the proliferation of BCPAP cells co-transfected with CRNDE overexpression vector (or empty vector) and miR-384 mimics (or mimics NC). **(B)** Colony formation analysis was performed to examine the cell growth of BCPAP cells co-transfected with CRNDE overexpression vector (or empty vector) and miR-384 mimics (or mimics NC). **(C)** Transwell invasion and **(D)** transwell migration assays were performed to examined the cell invasion and migration of BCPAP cells co-transfected with CRNDE overexpression vector (or empty vector) and miR-384 mimics (or mimics NC). N = 4, ^*^P<0.05.

To further determine if the effects of CRNDE is dependent on miR-384, the rescue experiments were performed. Transfection with miR-384 mimics alone significantly suppressed cell proliferation, decreased the number of colonies, the number of invaded and migrated cells in BCPAP cells compared with Mimics NC group (Supplementary Figure 1). Co-transfection with CRNDE overexpressing vector and mimics NC enhanced cell proliferation (Figure 6A), increased the number of colonies (Figure 6B), the number of invaded cells (Figure 6C) and the number of migrated cells (Figure 6D) compared with co-transfection with empty vector and mimics control; while co-transfection with CRNDE overexpressing vector and miR-384 mimics significantly attenuated cell proliferation, cell growth and cell invasion/migration compared with co-transfection with CRNDE overexpressing vector and mimics NC (Figure 6).

### PTN is a target of miR-384

**Figure 7:**
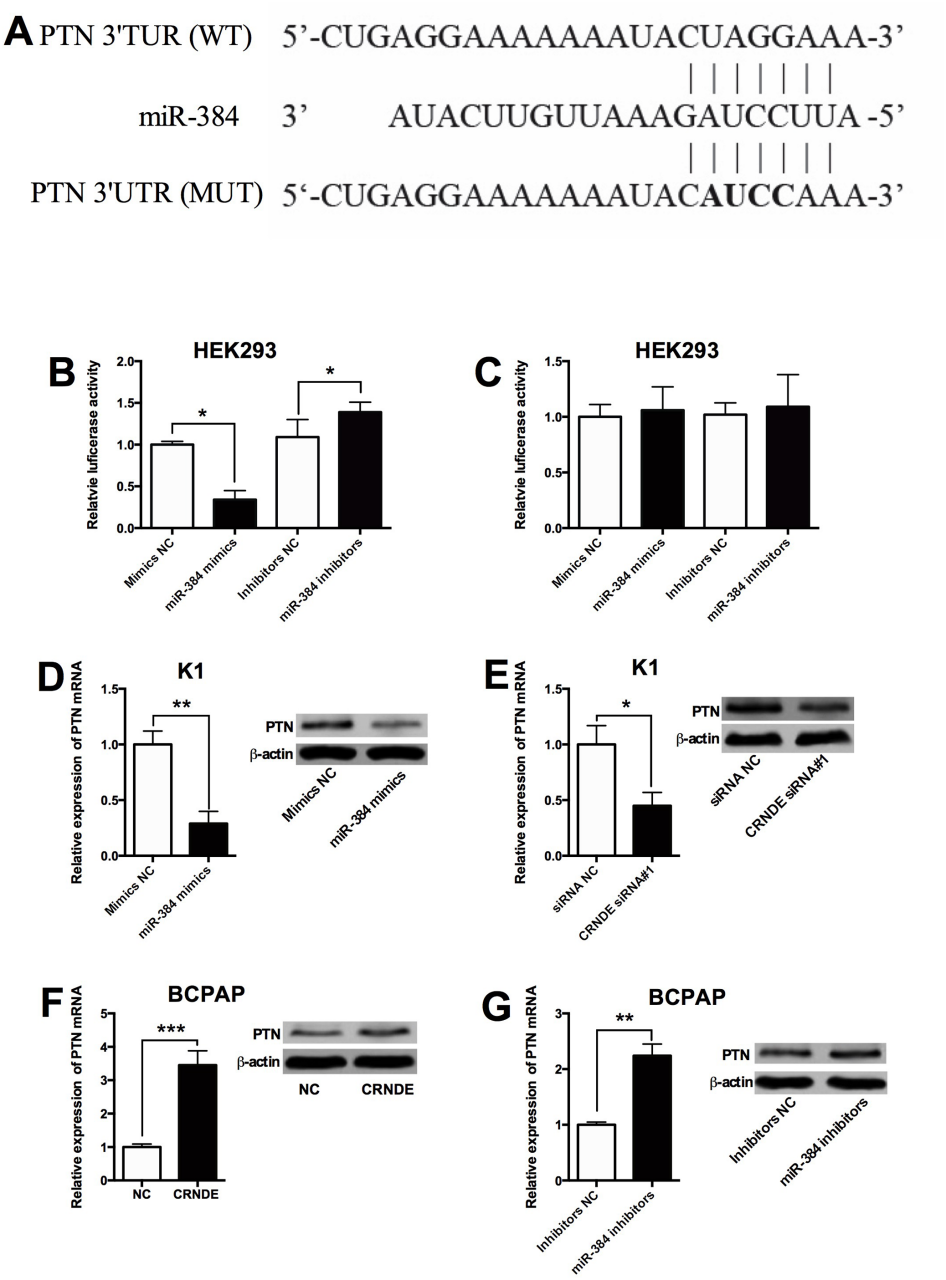
PTN is a target of miR-384 **(A)** The seed sequence of miR-384 matched the sequence of PTN 3'UTR. The sequences include the wild type (WT) and mutated (MUT) target sites, the mature miR-384 and the target sequence bound by miR-384. **(B)** Luciferase report assay of HEK293 cells co-transfected with PTN 3'UTR reporter plasmid containing WT sequences and miR-384 mimics or miR-384 inhibitors, or their corresponding negative controls i.e. mimics NC and inhibitors NC, and luciferase reporter activity was suppressed by miR-384 mimics transfection and increased by miR-384 inhibitors transfection. **(C)** Luciferase report assay of HEK293 cells co-transfected with PTN 3'UTR reporter plasmid containing MUT sequences and miR-384 mimics or miR-384 inhibitors, or their corresponding negative controls, and luciferase reporter activity was not affected by miR-384 mimics transfection and miR-384 inhibitors transfection. **(D)** The K1 cells transfected with miR-384 mimics showed an increased expression of PTN mRNA and protein compared with control group (Mimics NC). **(E)** The K1 cells transfected with CRNDE siRNA showed a decreased expression of PTN mRNA and protein compared with control group (siRNA NC). **(F)** The BCPAP cells transfected with CRNDE overexpressing vector showed an increased expression of PTN mRNA and protein in BCPAP cells compared with control group (NC). **(G)** The BCPAP cells transfected with miR-384 inhibitors showed an increased expression of PTN mRNA and protein compared with control group (Inhibitors NC). N = 4, ^*^P<0.05, ^**^P<0.01, ^***^P<0.001.

As miRNAs were found to regulate gene expressions via targeting the 3'UTR, the targets of miR-384 were predicted by Targetscan, and we found that PTN was a potential target of miR-384 (Figure 7A). In order to further confirm the interaction between miR-384 and PTN, the wild type 3'UTR of PTN and mutated 3'TUR of PTN reporter vectors were constructed to perform the luciferase reporter assay. As shown in Figure 7B, miR-384 mimics transfection reduced the luciferase activity of wild type 3'UTR of PTN reporter vector, while miR-384 inhibitors transfection enhanced the luciferase activity of wild type 3'TUR of PTN reporter vector; while miR-384 mimics transfection or miR-384 inhibitors transfection had no significant effect on the luciferase activity of mutated 3'UTR of PTN reporter vector (Figure 7C). In addition, miR-384 mimics transfection in K1 cells suppressed the expression of of PTN mRNA and protein compared with control group (Figure 7D); and knock-down of CRNDE suppressed the expression of PTN mRNA and protein in K1 cells (Figure 7E). On the other hand, overexpression of CRNDE increased the expression of PTN mRNA and protein in BCPAP cells compared with control group (Figure 7F), and miR-384 inhibitors transfection in BCPAP cells increased the expression of PTN mRNA protein compared with control group (Figure 7G).

**Figure 8:**
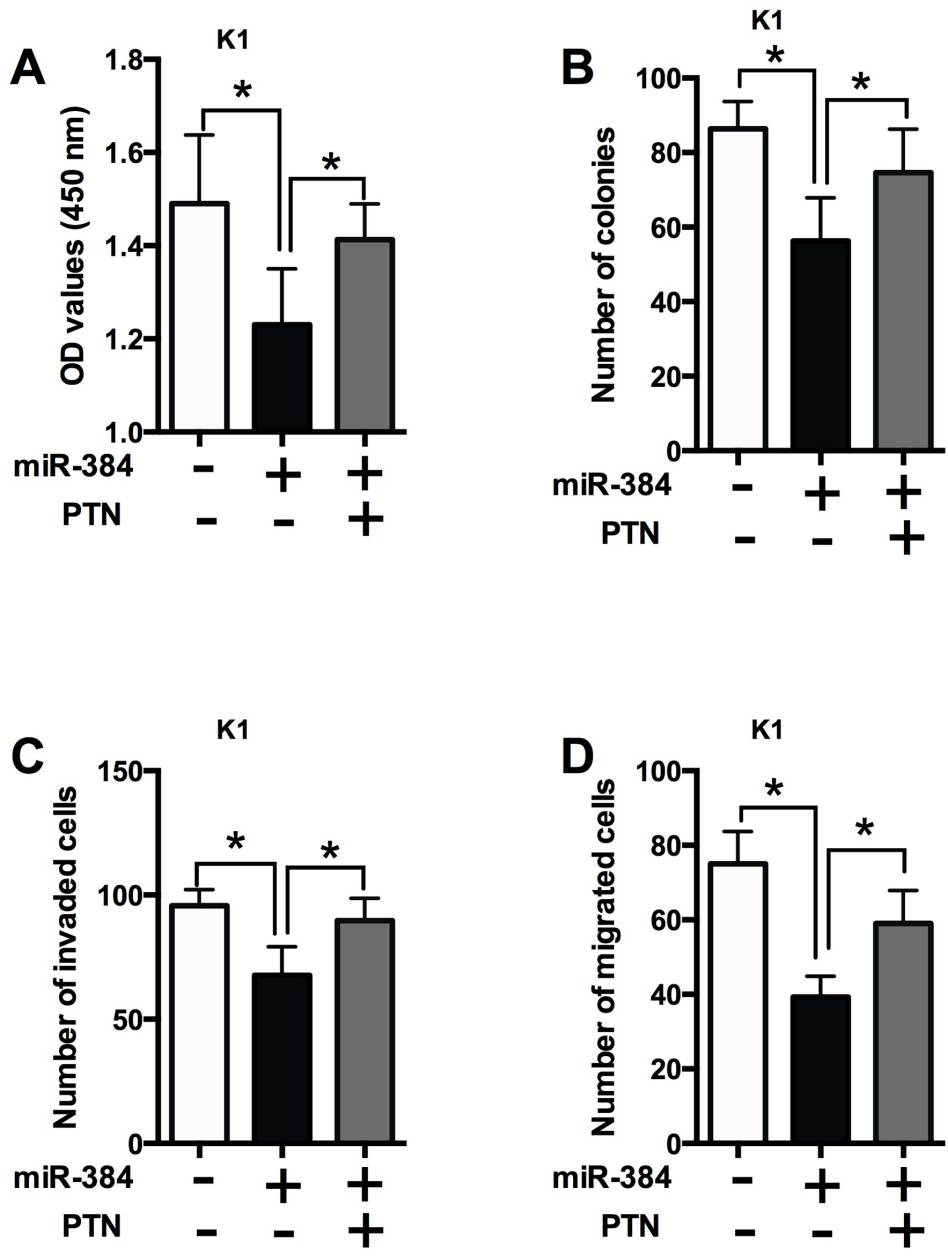
The effects of miR-384 in PTC cells is dependent on PTN **(A)** CCK-8 assay analysis was performed to examine the proliferation of K1 cells co-transfected with miR-384 mimics (or mimics NC) and PTN overexpressing vectors (or empty vectors). **(B)** Colony formation analysis was performed to examine the cell growth of K1 cells co-transfected with miR-384 mimics (or mimics NC) and PTN overexpressing vectors (or empty vectors). **(C)** Transwell invasion and **(D)** transwell migration assays were performed to examined the cell invasion and migration of K1 cells co-transfected with miR-384 mimics (or mimics NC) and PTN overexpressing vectors (or empty vectors). N = 4, ^*^P<0.05.

To further determine if the effects of miR-384 is dependent on PTN, the rescue experiments were performed. Co-transfection with miR-384 mimics and empty vector suppressed cell proliferation (Figure 8A), decreased the number of colonies (Figure 8B), the number of invaded cells (Figure 8C) and the number of migrated cells (Figure 8D) compared with co-transfection with empty vector and mimics control; while co-transfection with miR-384 mimics and PTN overexpressing significantly promoted cell proliferation, cell growth and cell invasion/migration compared with co-transfection with miR-384 mimics and empty vector (Figure 8).

### Expression of PTN in PTC tissues and its correlation with miR-384 and CRNDE

**Figure 9:**
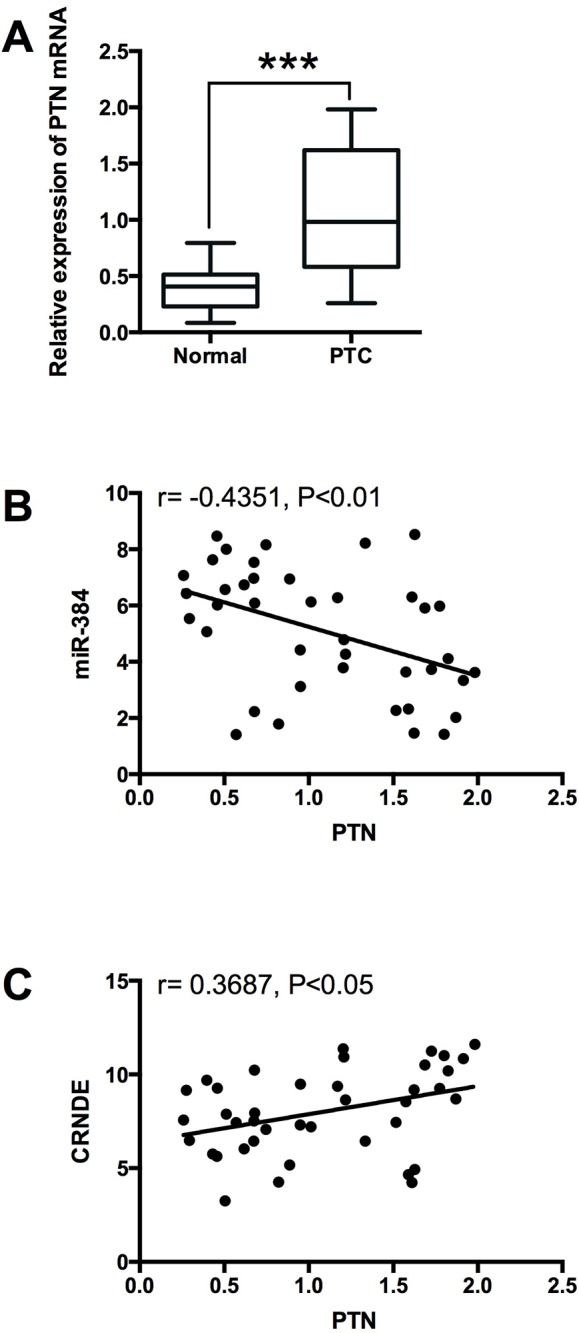
Expression of PTN in PTC tissues and its correlation with miR-384 and CRNDE **(A)** Analysis of 40 paired tumor tissue samples (adjacent non-tumor tissue samples and tumor tissues) showed that the expression of PTN was increased in tumor tissues (PTC) compared with adjacent normal tissues (n = 40), ^***^P<0.001. **(B)** Spearman correlation analysis showed that PTN was inversely correlated with miR-384 in PTC tissues, r = -0.4351, P<0.01, and **(C)** PTN was positively correlated with CRNDE in PTC tissues, r = 0.3687, P<0.05.

To examine the expression of PTN in PTC tissues, we performed qRT-PCR experiments to determine the expression of PTN mRNA in 40 adjacent normal thyroid tissues and 40 PTC tissues, and PTN in the PTC tissues was up-regulated compared with adjacent normal tissues (Figure 9A). The Spearman correlation analysis showed that PTN mRNA expression was inversely correlated with miR-384 expression in PTC tissues (Figure 9B), and positively correlated with CRNDE expression in PTC tissues (Figure 9C).

## DISCUSSION

In the present study, the main findings showed CRNDE was up-regulated in the PTC tissues and PTC cell lines, and overexpression of CRNDE promoted PTC cell proliferation, invasion and migration, and down-regulation of CRNDE exerted suppressive effects on PTC cell proliferation, invasion and migration. Further mechanistic studies highlighted the important roles of CRNDE/miR-384/PTN axis in the progression of PTC, in which CRNDE promotes cell proliferation, invasion and migration at least partly via competitively binding miR-384 in papillary thyroid cancer.

CRNDE was first identified in the colorectal adenomas and adenocarcinomas, and the functional role of CRNDE was demonstrated in various types of cancers. CRNDE was found to promote cell growth and invasion through mTOR signaling in glioma [11], and CRNDE also promoted tumor growth in medulloblastoma [27]. In the gallbladder cancer, CRNDE acted as a scaffold of DMBT1 and C-IAP1 complexes to activate PI3K-AKT pathway, which subsequently promoted gallbladder carcinoma carcinogenesis [12]. CRNDE can also activate Wnt/β-catenin signaling pathway in human breast cancer [13]. In the thyroid cancer, CRNDE was found to be up-regulated in the PTC tissues and CRNDE was associated with poor prognosis in PTC patients [15]. In agreement with previous studies, we demonstrated the up-regulation of CRNDE in PTC tissues. *in vitro* functional assays showed that overexpression of CRNDE promoted cell proliferation, invasion and migration, while down-regulation of CRNDE suppressed cell proliferation, invasion and migration in PTC cell lines, suggesting that CRNDE may play an oncogenic role in PTC.

The underlying mechanisms of lncRNAs regulating cancer progression may be via targeting the downstream miRNAs. In the breast cancer, CRNDE acted as a molecular sponge of miR-136 to promote cancer progression [13]. CRNDE also promoted colorectal cancer cell proliferation and chemoresistance via interacting with miR-181a-5p [28]. In gastric cancer, CRNDE promoted cancer progression via targeting miR-145 [11]. In the present study, the bioinformatics analysis revealed the potential interaction between CRNDE and miR-384, which was confirmed by luciferase reporter assay. Overexpression of CRNDE suppressed the expression of miR-384, and down-regulation of CRNDE increased the expression of miR-384 in PTC cell lines, and enforced expression of miR-384 attenuated the oncogenic effects of CRNDE, suggesting that CRNDE acted as a molecular sponge of miR-384 to promote cancer progression in PTC. Consistently, CRNDE promoted hepatic carcinoma cell proliferation, invasion and migration by sponging miR-384 [10], and CRNDE also promoted malignant progression of glioma by attenuating miR-384/PIWIL4/STAT3 axis [24]. The functional role of miR-384 was shown in glioma [24], hepatocellular carcinoma [25], and colorectal cancer [26], and miR-384 suppressed the cancer progression in these cancers. In our study, we showed that miR-384 was down-regulated in PTC tissues and cell lines, and overexpression of miR-384 increased PTC cell proliferation, invasion and migration, while knock-down of miR-384 exerted suppressive effects. Collectively, these results may imply that miR-384 was tumor-suppressive in PTC. Apart from functional role of CRNDE as a competitive endogenous RNA, CRNDE also played the oncogenic role via acting a scaffold of DMBT1 and C-IAP1 complexes to activating PI3K-AKT pathway in gallbladder cancer [12]. In addition, the histone acetylation in the promoter region of CRNDE accounted for the CRNDE up-regulation, and the level of CRNDE expression could be regulated by mammalian Target of Rapamycin signaling in glioma [29]. Due to the effects of CRNDE on diverse signaling pathways, further studies may be performed to fully understand the CRNDE/miR-384/PTN in PTC progression.

In the present study, PTN was predicted as a downstream target of miR-384, which was confirmed by luciferase reporter assay. PTN, also known as heparin-binding growth-associated molecule, is a 136-amino acid secreting growth/differentiation cytokine that is developmentally regulated [30]. PTN was found to be up-regulated in various types of human cancers, and overexpression of PTN was suggested to promote cell proliferation and angiogenesis, contributing to cancer progression [31–33]. Recent study also demonstrated the concentration in the fine-needle aspiration thyroid tissues was increased PTC [34]. Mechanistically, PTN was found to exert its functional role via interacting with cell surface proteoglycans, or via binding to its selective cell surface receptor, protein tyrosine phosphatase receptor Z1 (PTPRZ1) [35], which results in the accumulation of tyrosine phosphorylation of multiple downstream proteins including calmodulin, β-catenin, and SRC kinase [35–37]. The phosphorylation of these downstream proteins has been suggested to be related to the activation activation of multiple pro-tumorigenic signaling cascades, which in turn promote cancer cancer progression [38]. Consistently, our results showed that PTN was up-regulated in PTC tissues and the mRNA expression level of PTN was negatively correlated with miR-384 expression, but positively correlated with CRNDE expression in PTC tissues. In addition, PTN was negatively regulated by miR-384 in PTC cells, and overexpression of PTN attenuated the tumor-suppressive effects of miR-384 in PTC cells. Taken together, these results may suggest that miR-384 regulated the PTC progression via targeting PTN.

In conclusion, our results demonstrated the oncogenic role of CRNDE in PTC and regulatory role of CRNDE in PTC progression may be partly via CRNDE/miR-384/PTN axis. However, the down-stream targets of CRNDE were not limited to miR-384 and further studies may examine other potential target of CRNDE to full elucidate the role of CRNDE in PTC progression.

## MATERIALS AND METHODS

### Patients and thyroid tissues collection

PTC tissues and paired adjacent noncancerous thyroid tissues were collected from 40 patients diagnosed with thyroid cancer from Jan 2014 to Dec 2016 at Shaoxing People's Hospital. All the patients had no chemotherapy or radiotherapy before surgery. All the studies were approved by the Ethical Committee of Shaoxing People's Hospital and informed consent was obtained from all patients.

### Cell lines and cell culture

Human thyroid normal cell line, Nthy-ori 3-1, PTC cell lines (BCPAP, KTC-1, and K1), and HKE 293 cells were purchased from ATCC company (ATCC, Manassas, USA). All the cells were cultured in the Dulbecco's modified Eagles' medium (DMEM, Invitrogen, Carlsbad, USA) supplemented with 10% fetal bovine serum (FBS, Invitrogen), and maintained in a humidified incubator with 5% CO_2_ at 37°C.

### Oliogoribonucleotides synthesis, plasmid constructs, and cell transfection

MiR-384 mimics, miR-384 inhibitors and the respective negative control (NC) miRNAs (mimics NC, and inhibitors), and CRNDE siRNA (CRNDE siRNA#1 and CRNDE siRNA#2) and the NC (siRNA NC) were purchased from Ribobio (Guangzhou, China). The CRNDE overexpressing vectors (pcDNA3.1-CRNDE) and the empty vector (pcDNA3.1), and the PTN overexpressing vectors (pcDNA3.1-PTN) and the empty vector (pcDNA3.1) were purchased from GeneRay (Shanghai, China). The miRNAs, siRNAs or plasmids were transfected/co-transfected by using Lipofectamine 2000 reagent (Invitrogen) according to the manufacturer's instructions. At 48 h after transfection/co-transfection, cells were subjected to further experimentation.

### RNA extraction quantitative real-time PCR (qRT-PCR)

Total RNA was isolated from cell lines and patient tissues by using TRIzol reagent (Invitrogen) according to the manufacturer's instructions. RNA samples were reverse-transcribed by Prime Script^TM^ RT reagent kit (Takara, Dalian, China). Real time PCR was performed on an ABI7500 real-time PCR system (Foster City, USA) using SYBR Premix Ex Taq^TM^ (Takara). U6 was used as an internal control for miR-384 and GAPDH was used as internal control for CRNDE and PTN.

### CCK-8 assay

The transfected cells were seeded in 96-well plates and at 0 h, 24 h, 48 h and 96 h post-treatment, 10 μL of CCK-8 solution (Beyotime, Beijing, China) was added into each well, followed by incubation for 4 h according to the manufacturer's instructions. The OD values was measured at 450 nm using a microplate reader (Bio-Tek, Winooski, USA).

### Colony formation assay

The transfected cells were seeded in 6-well plates at a density of ∼500 cells/well and incubated with DMEM medium with 10% FBS at 37°C. Two weeks after seeding, the cells were fixed and stained with 0.1% crystal violet, and the number of colonies were examined under a light microscope.

### Transwell invasion and migration assays

Cells were re-suspended in 100 μL serum-free medium and were plated in the top chamber of each insert (8-μm pore size, Corning, USA) with a Matrigel-coated membrane (BD Bioscience, San Jose, USA) for the transwell invasion assay and a non-Matrigel-coated membrane for the Transwell migration assay. Lower chambers of the inserts were filled with DMEM medium with 10% FBS. After 12 h of incubation, cells that invaded/migrated to the lower surface of the insert were fixed, stained, and counted under a light microscope.

### Western blot assay

Proteins from cells were extracted by using the lysis RIPA buffer. The extracted proeins were then subjected to 10% SDS-PAGE electrophoresis and transferred onto the PVDF membranes. The membranes were blocked with 5% skimmed milk at room temperature for 1 h, and then the membranes were incubated overnight at 4°C with the following primary antibodies: anti-PTN (1:1000, Abcam, Cambridge, UK) and β-actin (1:2000, Abcam), and the membranes were incubated with HRP-conjugated secondary antibodies (Abcam) for 1 h at room temperature. The bands were visualized by using the ECL kit (Thermo Fisher Scientific, Rockford, USA).

### Luciferase reporter assay

HEK293 cells seeded in 24-well plates, and HEK293 cells were co-transfected with pmirGLO-CRNDE-WT, or pmirGLO-CRNDE-MUT, or pmirGLO-3'UTR of PTN-WT, or pmirGLO-3'UTR of PTN-MUT and miR-384 mimics, or miR-384 inhibitors, or the respective miRNA NCs. The transfection was performed by using the Lipofectamine 2000 reagent (Invitrogen). Luciferase activities were measured 48h after transfection using the dual-luciferase reporter assay system (Promega, Madison, USA).

### Statistical analysis

All the graphs-plotting and data analysis were performed by using the GraphPad Prism Version 6.0 software. All the data were shown as mean ± standard deviation. The significant differences between different groups were analyzed by t-test (comparison for two groups) or one-way ANOVA (comparison for more than two groups). The relationship between miR-384 and its target genes were analyzed by the Spearman's correlation analysis. P<0.05 was considered to be statistically significant.

## SUPPLEMENTARY MATERIALS FIGURE


