# Supplementary material for: LncRNA CRNDE promotes cell proliferation, invasion and migration by competitively binding miR-384 in papillary thyroid cancer
Source: Oncotarget. 2017 Nov 30;8(66):110552–65. doi: 10.18632/oncotarget.22819 (PMC5746403; doi:10.18632/oncotarget.22819)
Supplement: Supplementary file 1 [file oncotarget-08-110552-s001.pdf]

LncRNA CRNDE promotes cell proliferation, invasion and migration by competitively binding miR-384 in papillary thyroid cancer

SUPPLEMENTARY MATERIALS

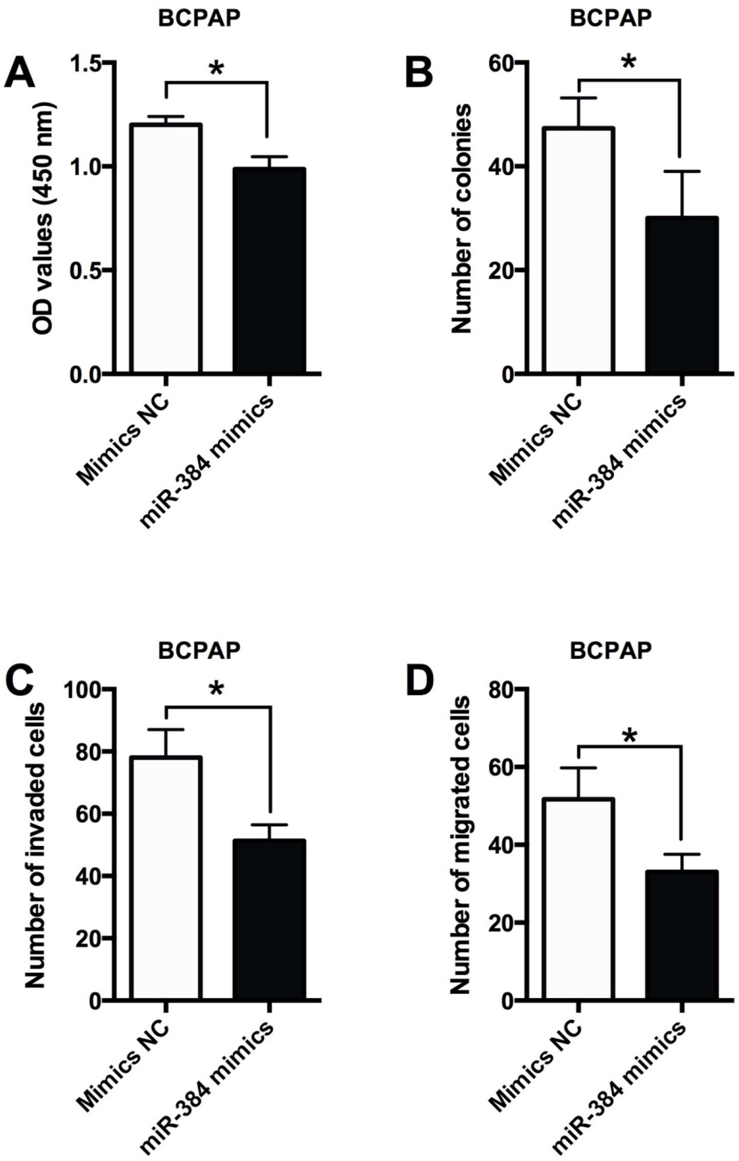

Supplementary Figure 1: Effects of miR-384 overexpression on the proliferation and invasion/migration in BCPAP cells. **(A)** MiR-384 mimics transfection in BCPAP cells suppressed cell proliferation compared with control group (Mimics NC) as measured by CCK-8 assay. **(B)** BCPAP cells transfected with miR-384 mimics showed a decreased growth ability compared with control group (Mimics NC) as measured by colony formation assay. **(C)** Overexpression of miR-384 decreased the number of invaded BCPAP cells compared with control group (Mimics NC) as measured by transwell invasion assay. **(D)** BCPAP cells transfected with miR-384 mimics had a decrease in the migrated cells compared with control group (Mimics NC) as measured by transwell migration assay. N = 4, $^{*}P<0.05$.
